# Gel wax-based tissue-mimicking phantoms for multispectral photoacoustic imaging

**DOI:** 10.1364/BOE.9.001151

**Published:** 2018-02-15

**Authors:** Efthymios Maneas, Wenfeng Xia, Olumide Ogunlade, Martina Fonseca, Daniil I. Nikitichev, Anna L. David, Simeon J. West, Sebastien Ourselin, Jeremy C. Hebden, Tom Vercauteren, Adrien E. Desjardins

**Affiliations:** 1 Wellcome / EPSRC Centre for Interventional and Surgical Sciences, University College London, Charles Bell House, 67-73 Riding House Street, London W1W 7EJ, UK; 2 Department of Medical Physics and Biomedical Engineering, University College London, Gower Street, London WC1E 6BT, UK; 3 Translational Imaging Group, Centre for Medical Image Computing, Department of Medical Physics and Biomedical Engineering, University College London, Gower Street, London WC1E 6BT, UK; 4 Institute for Women’s Health, University College London, 86-96 Chenies Mews, London WC1E 6HX, UK; 5 Department of Development and Regeneration, KU Leuven (Katholieke Universiteit), Belgium; 6 Department of Anaesthesia, University College Hospital, Main Theatres, Maple Bridge Link Corridor, Podium 3, 235 Euston Road, London NW1 2BU, UK

**Keywords:** (170.3880) Medical and biological imaging, (110.5120) Photoacoustic imaging, (170.7170) Ultrasound, (110.3000) Image quality assessment, (160.4760) Optical properties, (120.5820) Scattering measurements

## Abstract

Tissue-mimicking phantoms are widely used for the calibration, evaluation and standardisation of medical imaging systems, and for clinical training. For photoacoustic imaging, tissue-mimicking materials (TMMs) that have tuneable optical and acoustic properties, high stability, and mechanical robustness are highly desired. In this study, gel wax is introduced as a TMM that satisfies these criteria for developing photoacoustic imaging phantoms. The reduced scattering and optical absorption coefficients were independently tuned with the addition of TiO_2_ and oil-based inks. The frequency-dependent acoustic attenuation obeyed a power law; for native gel wax, it varied from 0.71 dB/cm at 3 MHz to 9.93 dB/cm at 12 MHz. The chosen oil-based inks, which have different optical absorption spectra in the range of 400 to 900 nm, were found to have good photostability under pulsed illumination with photoacoustic excitation light. Optically heterogeneous phantoms that comprised of inclusions with different concentrations of carbon black and coloured inks were fabricated, and multispectral photoacoustic imaging was performed with an optical parametric oscillator and a planar Fabry-Pérot sensor. We conclude that gel wax is well suited as a TMM for multispectral photoacoustic imaging.

## 1. Introduction

Tissue-mimicking phantoms are widely used for evaluating new medical imaging systems and for training clinical practitioners. Traditionally, phantoms for optical modalities have been developed separately from those for ultrasonic modalities. For optical imaging modalities such as diffuse optical tomography and fluorescence imaging, controlling optical properties such as optical scattering and absorption coefficients has been important. Likewise, for ultrasound imaging, speed of sound, acoustic attenuation and acoustic impedance are often the most relevant parameters being looked at in phantoms. Hybrid imaging and sensing modalities, which rely on the propagation of both optical and ultrasound waves, present new challenges for phantom development. Photoacoustic (PA) imaging is a prominent example: ultrasound waves are generated with pulsed or modulated light [1–3]. For this modality, it is necessary to develop tissue-mimicking materials (TMMs) with optical and acoustic properties that can be independently tuned within physiological ranges. Ideally, these TMMs would also have good temporal stability and mechanical robustness.

Existing TMMs that are widely used for optical and ultrasound phantoms have limitations for photoacoustic imaging. Aqueous phantoms such as those based on agar [4] and gelatin [5] are often useful, since constituents to modify optical and acoustic properties are well known [6–8]. However, they tend to have limited mechanical strength and high evaporation rates. Polyester resin [9], epoxy resin [10], and silicone [11] are often used for optical imaging, but they have unrealistic sound speeds and/or high acoustic attenuation values [12,13]. Likewise, polyurethane and condensed milk gel are used for commercial ultrasound phantoms [14], but their high intrinsic optical absorption and/or scattering limited their use for optical imaging phantoms [14,15]. Poly(vinyl alcohol) (PVA) has been used successfully for ultrasound and photoacoustic imaging [16–19]. Its optical scattering, speed of sound, acoustic attenuation, and Young’s modulus increases with the number of freeze-thaw cycles performed during the phantom fabrication process, so that it is not necessary to include additional optical and acoustic scatterers. However, the fabrication process is rather time consuming. Dissolving PVA in a mixture of water and dimethyl sulfoxide (DMSO) can yield optically transparent and mechanically robust gels to which optical absorbers and scatterers can be added [17,18]. Polyvinyl chloride plastisol (PVCP) is also used for photoacoustic imaging phantoms [20,21]. Its optical scattering and absorption can be well controlled by adding optical scatterers and inks [22–25]. However, PVCP has higher acoustic attenuation than that of some fatty tissues, even in its native form [22–26].

Mineral oil-based materials have shown promise as TMMs [27–32]. A study by Cabrelli *et al.* [29] reported that the optical and acoustic properties of mineral oil can be varied by adding styrene-ethylene/butylene-styrene copolymer and low-density polyethylene. The measured acoustic properties of their mineral oil-based compositions were within the range of values reported for soft tissues. However, in that study, the optical absorption and scattering coefficients were not independently tuned and the values were lower than those for typical soft tissues. Gel wax is an optically transparent mineral-oil based material that has been widely used in the candle industry. Recently, it was used as a TMM for ultrasound-guided needle biopsy phantoms [27] and for optical imaging phantoms [30,31]. In this study, gel wax-based compounds are introduced as a TMM for photoacoustic imaging for the first time. The optical properties were tuned with the addition of TiO_2_ and coloured inks. Optical and acoustic characterisation was performed on homogeneous phantoms. The potential of gel wax for use as a TMM in multispectral photoacoustic imaging was demonstrated with optically heterogeneous phantoms.

## 2. Materials and methods

### 2.1. Gel wax material and slab fabrication

**Fig. 1:**
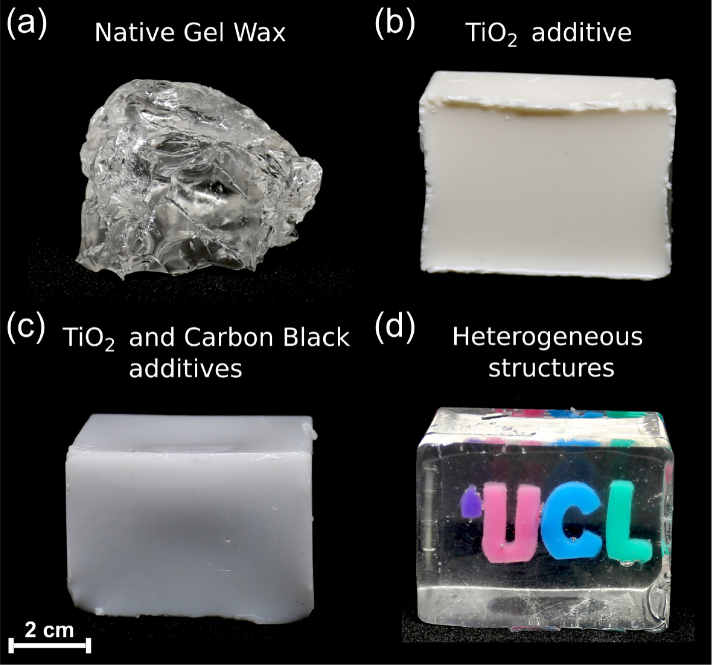
Photographs of gel wax phantoms with different compositions. (a) Optically transparent native gel wax, prior to processing. (b) Native gel wax with TiO_2_ added for optical scattering. (c) Native gel wax and TiO_2_, with carbon black ink added for optical absorption. (d) The University College London (UCL) logo fabricated with inclusions that comprised TiO_2_ and differently-coloured inks in a native gel wax background ( Visualization 1).

Native gel wax (FF1 003, Mindsets Online, Waltham Cross, UK; Fig. 1(a)

) was first melted to incorporate additives to form compositions with different optical properties. It was heated to a liquid state at 200 °C, and additives were gradually added with continuous mechanical mixing (IKA Eurostar 20 High Speed Digital, supplied from Fisher Scientific, Pittsburgh, PA) until the solution was visually homogeneous (*ca*. 60 min). Subsequently, this solution was sonicated (CL18, Fisher Scientific, Pittsburgh, PA; 120 W) for 1 min (65% of maximum power) for further homogenization. After sonication, the solution was reheated to 200 °C, and then degassed for approximately 2 min. The sonication-degassing steps were repeated until air bubbles were absent with visual inspection. Each degassing step was limited to less than 2 min to maintain the mobility of the solution, so that bubbles can migrate to its surface. To tune optical scattering, TiO_2_ (13463-67-7, ReagentPlus 99%, Sigma-Aldrich, Germany) was used as an additive (Fig. 1(b)). To tune optical absorption, oil-based inks were used: carbon black (Fig. 1(c)), green, red, blue and violet (BKC 1860, GRC 43104, RDC 63827, BLC 25291, VLC 71139; Caligo safe wash relief inks, Cranfield Colours, Cwmbran, UK).

Gel wax-based compound slabs were fabricated for measurements of optical and acoustic properties (Sec. 2.3 – 2.4), of photoacoustic spectroscopy, and of photostability (Sec. 2.5). Melted gel wax compositions with additives were poured into rectangular moulds. These moulds were created with an acrylic spacer (nominal thickness: 2 or 5 mm) positioned between two glass microscope slides. This spacer had an opening that allowed for the mixture to be poured in between the two slides. After the compound was cooled at room temperature, the glass slides were removed, so that a rectangular gel wax slab (*ca.* 35 × 50 mm) surrounded by the acrylic spacer remained.

### 2.2. Heterogeneous phantom fabrication

Two series of vessel phantoms comprising cylindrical inclusions with different ink concentrations were fabricated for multispectral photoacoustic imaging (Sec. 2.6). In the first series, the gel wax-based inclusions contained 0.02, 0.05 and 0.10 w/v% carbon black ink. In the second series, they contained 0.05 w/v% coloured inks. To introduce scattering, 0.10 w/v% TiO_2_ was incorporated into both the inclusions and the gel wax background. The background did not contain ink. The inclusions (diameter: 5 mm) were first cast using 3D printed cylindrical moulds. After solidification at room temperature, they were removed from the moulds and cut (length: 5 mm) with a scalpel. These cylinders were then placed into the rectangular mould with the acrylic spacer (*c.f.* Sec. 2.1), with their flat ends sandwiched by the two glass microscope slides. The background mixture was poured into that mould at *ca.* 90 °C, so that it flowed around the cylinders, and the phantom was allowed to solidify at room temperature. Finally, the phantom was extracted and trimmed (final dimensions: 20 × 20 × 5 mm).

A 3D University College London (UCL) logo (Fig. 1(d)) was created as an additional demonstration of optically heterogeneous gel wax-based phantoms, with greater complexity than the vessel phantoms. The UCL logo comprised gel wax-based inclusions with four differently-coloured inks (0.05 w/v%) and TiO_2_ (0.05 w/v%). A phantom with this logo was created with 3 steps. First, the inclusions (thickness: 5 mm) were cast using a laser-cut acrylic mould and removed after cooling to room temperature. Second, they were positioned into a mould in the same manner as the cylindrical inclusions were incorporated into the vessel phantoms. Third, the resulting thin UCL logo slab was incorporated into native gel wax using a larger, rectangular mould (64 × 48 × 42 mm). Native melted gel wax was poured up to the middle of this mould, and then left to partially cool. The thin UCL logo slab was placed onto the partially cooled native gel wax and partially sunk into it. After solidification, the larger rectangular mould was completely filled with native melted gel wax. By visual inspection and manipulation, this phantom was found to have good temporal stability; over the duration of this study (*ca.* 1 year), spatial diffusion of these inks, and deformations and colour changes of the background native gel wax in the phantom were not visually apparent.

### 2.3. Measurements of optical properties

Three series of gel wax-based slabs with different concentrations of absorbers and scatterers were fabricated. In the first, scattering was held constant with TiO_2_ (0.10 w/v%); absorption was varied with carbon black ink (0.005 to 0.05 w/v%). In the second series, absorption was held constant with carbon black ink (0.005 w/v%); scattering was varied with TiO_2_ (0.050 to 0.125 w/v%). In the third series, coloured inks (0.005 w/v%) and TiO_2_ 0.10 w/v% were used for absorption and scattering, respectively.

To characterise the optical properties, each gel wax-based slab was cut into two pieces and measurements from both sides of each piece were obtained. A dual beam spectrophotometer (Lambda 750, Perkin Elmer, Waltham, USA) with a 100 mm integration sphere provided transmittance and reflectance measurements in a wavelength range of 400 to 1600 nm. These measurements were used as input parameters to the inverse adding-doubling (IAD) algorithm [33] to estimate the optical absorption (*μ_a_*) and the reduced scattering coefficient ( $\mu_{s}^{\prime }$). The IAD algorithm iteratively searches for a solution to the radiative transfer equation (RTE) under the assumption of layered samples with homogeneous optical properties and uniform light illumination. The scattering anisotropy factor (*g*) was assumed to be *g* = 0.6 and the refractive index for gel wax was set to 1.469 [31].

### 2.4. Measurements of acoustic properties

The acoustic properties of four gel wax-based slabs were characterized. The first slab did not have any additives. The second slab comprised only TiO_2_ (0.10 w/v%). The third and fourth slabs comprised both TiO_2_ (0.10 w/v%) and a black or blue ink (0.05 w/v%).

**Fig. 2:**
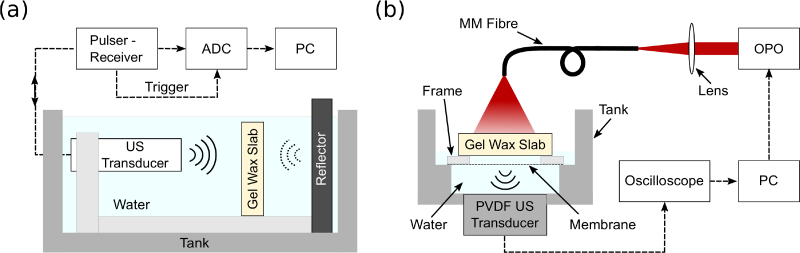
Schematics of the experimental setups used to characterise gel wax properties. (a) Setup to measure the speed of sound and acoustic attenuation of gel wax. An ultrasonic pulser/receiver was used to a drive an ultrasound (US) transducer and receive reflections from a metal reflector. Gel wax-based slabs of different thicknesses were positioned between the US transducer and the reflector. The received signals were digitized by an analog-to-digital converter (ADC) and then transferred to a personal computer (PC) for processing. (b) Setup to acquire photoacoustic signals from gel wax slabs for spectroscopic and photostability measurements. Each slab was mounted on top of a rectangular acrylic frame with a thin plastic membrane. Excitation light was delivered through a fibre-coupled Nd:YAG pumped optical parametric oscillator (OPO), and a thin polyvinylidene fluoride (PVDF) ultrasound transducer was used to receive the photoacoustically-generated ultrasound waves. Signals from the PVDF transducer and the photodiode were captured simultaneously with an oscilloscope and transferred to a PC for processing.

An insertion method [19] was used to measure the acoustic attenuation and the speed of sound of the gel wax compositions (Fig. 2(a)

). A single-piezo transducer (V312-SU, Olympus, Shinjuku, Japan), centred at 10 MHz, was operated in pulse-echo mode at 1 kHz repetition rate. The transducer was affixed on a 3D printed holder, with its acoustic axis perpendicular to the surface of a metal reflector. The ultrasound transducer and the reflector were mounted on a metal plate and immersed in deionized water at room temperature (*ca.* 20 °C). An ultrasonic pulser/receiver (5077PR, Olympus, Shinjuku, Japan) was used to drive the transducer and receive the echo signal from the reflector. This signal was digitised at 100 MS/s (USB-5132, National Instruments, Austin, USA) and then transferred to a PC for processing. The water temperature was recorded with a digital thermometer (4378, Fisher Scientific, Pittsburgh, UK).

For each gel wax composition, pulse-echo ultrasound signals (*S*_1_, *S*_2_) for two gel wax-based slabs (2 and 5 mm nominal thicknesses) were acquired separately. Acquisitions of *S*_1_ and *S*_2_ were repeated 5 times from distinct regions within the slabs. The speed of sound (*c_s_*) was estimated by solving for this variable in Equation (1):

(1) $$\Delta t=\frac{2(d_{2}-d_{1})}{c_{s}}-\frac{2(d_{2}-d_{1})}{c_{w}}$$

where *c_w_* is the tabulated value for the speed of sound of water [34], and *d*_1_ and *d*_2_ are the thicknesses of the sample pair as they were measured with a digital calliper. Here, Δt is the difference in the time-of-flights that was calculated using cross-correlation of *S*_1_ and *S*_2_. The acoustic attenuation of the sample, *α_s_*(*f*), was estimated as:

(2) $$\alpha_{s}(f)=\frac{10}{d_{2}-d_{1}}{\text{log}}_{10}\;\left[\frac{I_{1}(f)}{I_{2}(f)}\right]+\alpha_{w}(f)$$

where *I*_1_(*f*) and *I*_2_(*f*) are the power spectra of *S*_1_ and *S*_2_, and *α_w_*(*f*) is the frequency (*f*) dependent acoustic attenuation of water [34]. The acoustic attenuation in most materials can be described with power law of the *α_s_*(*f*) = *α*_0_ *f^n^*, in which *α*_0_ is termed the pre-factor, and *n* a positive exponent. To estimate *α*_0_ and *n*, a power law was fitted to the estimated values of using a non-linear least squares regression algorithm (Matlab, Mathworks, Natick, USA).

### 2.5. Measurements of photoacoustic spectroscopy and photostability

Four gel wax-based slabs, each comprising a different coloured ink (0.05 w/v%) and TiO_2_ (0.10 w/v%), were used to measure the photoacoustic spectra of these inks. The red ink was excluded due to its low optical absorption. Measurements were performed by photoacoustically exciting the slabs and directly receiving the generated ultrasound in transmission mode (Fig. 2(b)). Each slab was mounted on a rectangular acrylic frame (thickness: 2 mm) with a thin plastic membrane (thickness: 50 *μ*m), at the top of a water-filled tank [35, 36]. It was acoustically coupled to the membrane with water. Ultrasound reception was performed with a polyvinylidene fluoride (PVDF) transducer (active area: 10 mm × 10 mm × 50 *μ*m) at the bottom of the tank (distance to slab: 1 cm). Excitation light was delivered through a fibre-coupled Nd:YAG pumped optical parametric oscillator (OPO) (SpitLight 600, Innolas, Krailling, Germany) with a 6 ns pulse duration and a 30 Hz pulse repetition rate. The distal end of the fibre was positioned 1.5 cm away from the top surface of the gel wax slab. The wavelength range from 550 to 830 nm corresponded to the measured optical absorption spectra of the inks. The fluence varied from 3.4 to 8.8 mJ/cm^2^, depending on the excitation wavelength. A small portion of the excitation light was reflected to a photodiode between the OPO output and the fibre, to obtain a measure of the incident pulse energy and thereby to compensate for variations in pulse energy. Signals from the PVDF ultrasound transducer and the photodiode were digitized simultaneously. The photoacoustic spectra, defined as the maximum amplitudes of the photoacoustic signals across the measured range of wavelengths, were then normalised to the maximum peaks of the optical absorption spectra for direct comparison. To investigate the photostability of the inks, peak-to-peak amplitudes of the photoacoustic signals obtained with excitation at the peaks of the optical absorption spectra (violet: 550 nm; green: 660 nm; blue and black: 725 nm) were monitored over 20,000 pulses.

### 2.6. Multispectral photoacoustic imaging of vessel phantoms

Multispectral photoacoustic imaging of the vessel phantoms was performed with a planar Fabry-Pérot ultrasound sensor, which was described in detail by Zhang *et al.* [37]. Excitation light provided by an OPO (*c.f.* Sec 2.5) was delivered to the top of the phantoms (fluence range: 2.7 to 5.8 mJ/cm^2^). For the carbon black ink vessel phantom, wavelengths of 630, 800 and 1210 nm were used. Additional wavelengths of 550 and 725 nm that corresponded to the peaks of the optical absorption spectra (violet and red: 550 nm; blue: 725 nm) were used for the coloured ink vessel phantom. The recorded photoacoustic signals were reconstructed offline using a time-reversal algorithm implemented with the *k-Wave* Matlab toolbox [38]. The reconstructed photoacoustic images were displayed as a maximum intensity projections (MIPs) over a depth of 0.2 mm from the phantoms’ top surfaces. Finally, each MIP image was normalised to the incident pulse energy. To study the relationship between the photoacoustic signal amplitude and the carbon black ink concentration, a circular region of interest (ROI) with a diameter of 3 mm was defined for each inclusion, and the average photoacoustic signal amplitudes across each ROI were calculated.

## 3. Results

### 3.1. Optical properties

**Fig. 3:**
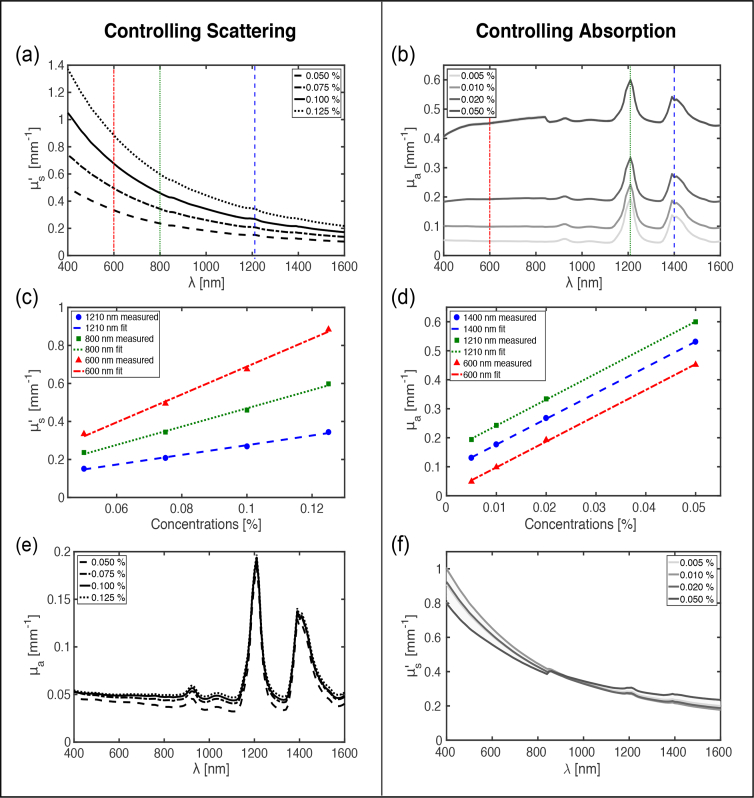
Measured reduced optical scattering and optical absorption coefficients of gel wax compounds with TiO_2_ and carbon black ink as additives. The two columns present how the optical scattering and absorption can be controlled. In the left column [(a),(c),(e)], the concentration of TiO_2_ was varied and the concentration of carbon black ink was held constant. In the right column [(b),(d),(f)], the concentration of carbon black ink was varied and the concentration of TiO_2_ was held constant. Each measured data point was obtained by averaging across 4 different spatial locations. Linear fitting was performed for the data in (c) and (d).

For gel wax samples with a constant concentration of carbon black ink, the estimated $\mu_{s}^{\prime }$ values generally increased with the concentration of TiO_2_ and decreased with wavelength (Fig. 3(a)

). Linear dependencies between $\mu_{s}^{\prime }$ and the TiO_2_ concentration were observed (R^2^ > 0.99; Fig. 3(c)). The corresponding estimated *μ_a_* values were found to be largely unchanged with TiO_2_ concentration, as expected (Fig. 3(e)).

Similarly, for gel wax samples with a constant concentration of TiO_2_, *μ_a_* generally increased with the carbon black ink concentration (Fig. 3(b)). Several prominent absorption peaks were present at 1210 and 1390 nm; there were additional peaks at 930 and 1040 nm (*c.f.* Fig. 3(e)). Taken together, these peaks can be attributed to the intrinsic absorption of native gel wax. Across the wavelength range of 400 to 900 nm, the absorption spectra were relatively flat. Linear dependencies between *μ_a_* and the carbon black ink concentration were observed (R^2^ > 0.99) (Fig. 3(d)). The corresponding $\mu_{s}^{\prime }$ values were found to be weakly dependent on the carbon black ink concentration, as expected (Fig. 3(f)).

**Fig. 4:**
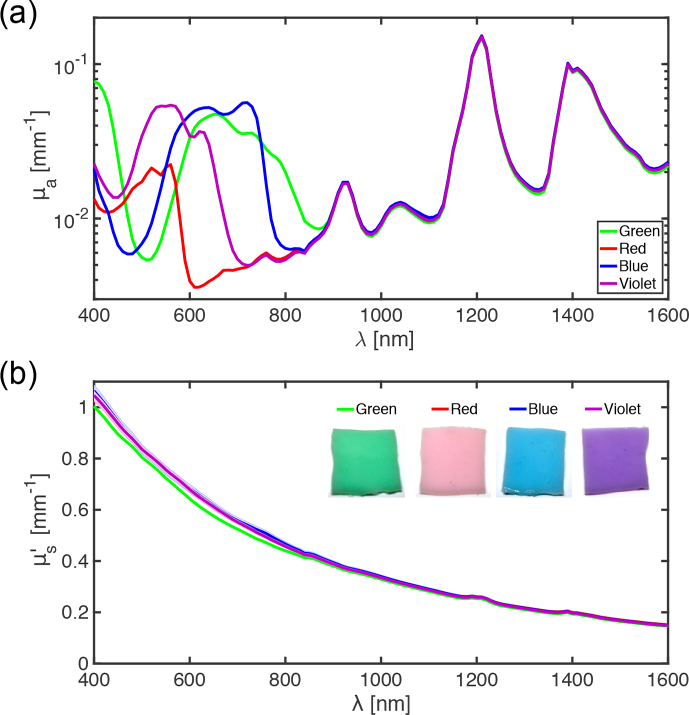
Spectroscopic measurements of optical absorption coefficients (a) and reduced scattering coefficients (b) for gel wax compositions with TiO_2_ and coloured inks (green, red, blue, and violet) as additives. For illustrative purposes, the photographs [inset in (b)] correspond to samples with ink concentrations that are 10 times higher than those of the measured samples.

For gel wax samples with different coloured inks as additives, distinct absorption spectra were present over the wavelength range of 400 to 900 nm (Fig. 4(a)

). These spectra differed in terms of their shapes and their maximum absorption peaks in this range (blue: 720 nm; violet: 560 nm; green: 650 nm, red: 560 nm). Across the range of 900 to 1600 nm, the peaks were highly consistent; here, absorption of the inks appeared to be very low. The $\mu_{s}^{\prime }$ values were very similar for all inks (Fig. 4(b)).

### 3.2. Acoustic properties

**Fig. 5:**
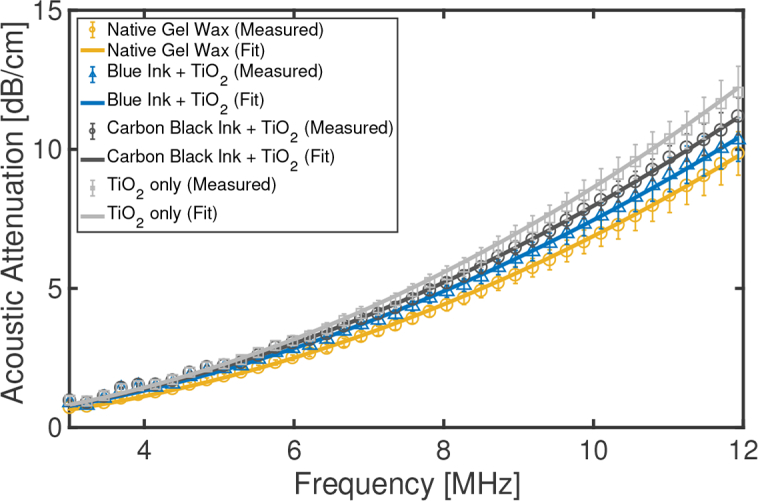
Measured acoustic attenuation of four gel wax compounds. Three of the compounds had additives; TiO_2_ was added to tune optical scattering and blue and carbon black inks were added to tune optical absorption. Fitting was performed with a frequency power law. Each measured data point was obtained by averaging across 5 different spatial locations (error bars: standard deviations).

The addition of TiO_2_ and inks had a modest effect on the acoustic properties of gel wax compounds (Fig. 5

). The frequency dependence of acoustic attenuation was well described with a power law. With the absence of additives, the attenuation [*α*(*f*) = (0.072 ± 0.008)*f*^(1.98±0.07)^ dB/cm] varied from 0.71 dB/cm at 3 MHz to 9.93 dB/cm at 12 MHz. Differences in acoustic attenuation across the gel wax compounds increased with frequency, with a maximum variation of 22% at 12 MHz. These differences may reflect high sensitivities to handling, positioning, and spatial variations in thickness of the gel wax-based slabs. The speed of sound was found similar across the measured gel wax compounds with mean ± standard deviation values of 1445 ± 2.7 m/s.

### 3.3. Photoacoustic spectroscopy and photostability

**Fig. 6:**
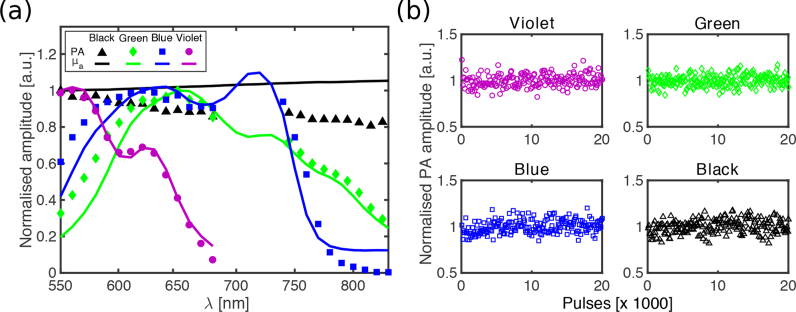
(a) Normalised photoacoustic spectra (symbols) and corresponding normalised optical absorption spectra (solid lines) for four gel wax compounds that contained carbon black ink and coloured inks (green, blue, and violet). Each compounds also contained TiO_2_. For each composition, spectra were normalised to the maximum value of the photoacoustic spectrum. (b) Normalised photoacoustic signal amplitudes from 20,000 excitation pulses, which indicated good photostability.

The photoacoustic spectra of gel wax-based samples with inks (2 mm nominal thickness) were in good agreement with the optical absorption spectra of the slabs within the measured range of 550 to 830 nm (Fig. 6(a)

). The spectra from the violet ink had the best agreement; those from the black ink diverged with increasing wavelength. All of the inks yielded stable photoacoustic signal amplitudes after 20,000 excitation light pulses (Fig. 6(b)).

### 3.4. Photoacoustic imaging

**Fig. 7:**
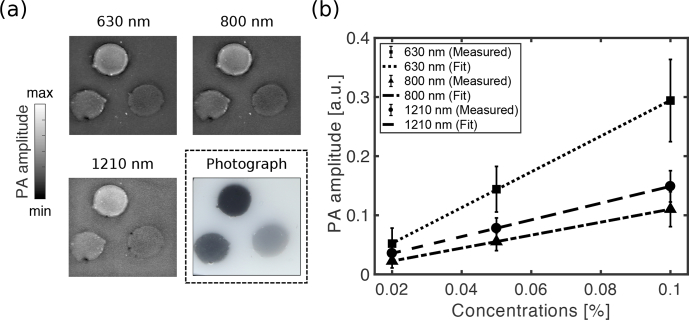
Photoacoustic imaging of the carbon black ink-based vessel phantom. The photoacoustic images, displayed as maximum intensity projections across a depth of 0.2 mm, were obtained with excitation wavelengths of 630, 800 and 1210 nm. A colour photograph of the phantom is also shown. The field of view is 20 × 20 mm. (b) Photoacoustic amplitudes from the vessel inclusions, averaged across circular region of interests (error bars: standard deviations), and linear fits.

With photoacoustic imaging of the heterogeneous carbon black ink phantom, all three inclusions with different concentrations were visible (Fig. 7(a)

). The PA image acquired at 1210 nm was more granular in appearance. This granularity can be attributed to the higher optical absorption of native gel wax at this wavelength, which led to greater visibility of small-scale spatial variations in the optical properties. At all wavelengths, the photoacoustic signal amplitude increased linearly (R^2^ > 0.9) with the carbon black ink concentration (Fig. 7(b)).

**Fig. 8:**
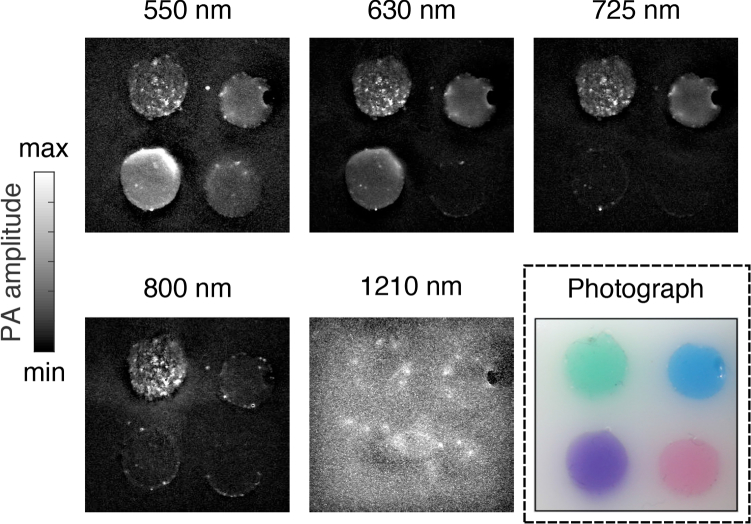
Photoacoustic imaging of the coloured ink vessel phantom with four inclusions: green (top left), blue (top right), violet (bottom left), and red (bottom right). The photoacoustic images, displayed as maximum intensity projections across a depth of 0.2 mm, were obtained with excitation wavelengths of 550, 630, 725, 800 and 1210 nm. A colour photograph of the phantom is also shown. The field of view is 20 × 20 mm.

With photoacoustic imaging of the heterogeneous phantom with coloured inks, the visibility of the inclusions depended on the excitation light wavelength (Fig. 8(a)

). For instance, at 550 nm, all four inclusions were visible, whereas at 800 nm, only the inclusion with the green ink was clearly visible. At 1210 nm, the inclusions could not be visually differentiated from the native gel wax background; indeed, the coloured inks were not significantly optically absorbing at this wavelength (*c.f.* Fig. 4(a)).

## 4. Discussion and conclusions

We introduced, for the first time, the use of gel wax as a mineral oil-based tissue-mimicking material (TMM) for photoacoustic imaging phantoms. Previous studies have shown that gel wax has favorable acoustic properties and mechanical robustness [27,32]. In this study, we reported that the reduced optical scattering and absorption coefficients could be tuned independently with TiO_2_ and oil-based inks, respectively. The wide range of reduced optical scattering coefficients that was achieved could be used to simulate different types of soft tissues [39, 40]. Whilst the inks had optical absorption spectra that were different from those of tissue chromophores, their prominent spectral variations, low diffusivity in gel wax, and high photostability could make them useful to evaluate spectral unmixing algorithms [41,42]. Additionally, we demonstrated that gel wax compounds can be used to create optically heterogeneous phantoms that are well suited to multispectral photoacoustic imaging in the wavelength range of 400 – 900 nm. The acoustic attenuation and speed of sound of the gel wax compositions were within the range of values reported for soft tissues [39].

Apart from the TiO_2_ powder, the materials used in this study were proprietary; their chemical compositions were not known in detail. For consistency across future studies, it would be valuable to have recipes for gel wax that are openly available to the imaging community. This is also the case for optical absorbers for dispersion in gel wax. With these developments, long term studies of the optical, acoustic, and mechanical stability will be important.

There are several ways in which the phantom creation methods presented in this study could be extended. To improve the optical homogeneity of coloured inclusions, absorbers and scatterers that are pre-dispersed in oil solutions with lower viscosity than the inks used here may be useful. The acoustic properties could be tuned by including additives such as glass beads, paraffin wax, and co-polymers, as demonstrated in previous studies [27–29, 32]. Moreover, these additives could be incorporated with different concentrations across the phantom to create acoustically heterogeneities. Although the phantom fabrication process involved repeated degassing steps in a low pressure chamber after heating and mixing of materials, several air bubbles were still present in the heterogeneous phantoms, particularly at the inclusion boundaries. To minimise bubble formation, mixing and pouring of gel wax compositions within low pressure chambers could be advantageous. For more anatomical realism, wall-less, fluid-filled vessels could be used [32,43], osseous structures could be incorporated with 3D printed polymers [44], and gel wax could even be directly 3D printed [31].

With controllable optical properties, favorable acoustic properties, temporal stability, and mechanical robustness, gel wax could be useful as a base TMM for many imaging and sensing modalities that involve light and/or ultrasound wave propagation. Additionally, it could be valuable as a spatially-localised constituent in photoacoustic imaging phantoms to mimic lipid rich tissue structures such as certain atherosclerotic plaques and nerves [45–48]. Gel wax has strong potential to become a next generation TMM for calibration, evaluation, and standardisation of imaging systems, and for clinical training.
